# Studying the Structure of Sodium Lauryl Ether Sulfate Solutions Using Dissipative Particle Dynamics

**DOI:** 10.1021/acs.jpcb.2c04329

**Published:** 2022-09-30

**Authors:** Rachel L. Hendrikse, Andrew E. Bayly, Peter K. Jimack

**Affiliations:** † School of Chemical and Process Engineering, University of Leeds, Leeds LS2 9JT, United Kingdom; ‡ EPSRC Centre for Doctoral Training in Fluid Dynamics at Leeds, University of Leeds, Leeds LS2 9JT, United Kingdom; § School of Computing, University of Leeds, Leeds LS2 9JT, United Kingdom

## Abstract

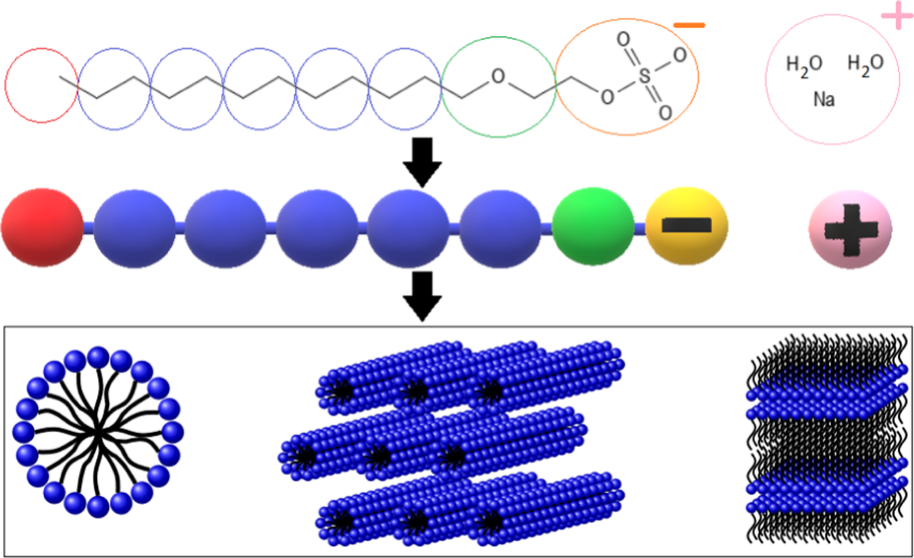

Sodium lauryl ether sulfate (SLES) is a common anionic surfactant used in a large number of personal care products. Commercial products typically contain a distribution in the number of ethoxy groups; despite this, there is limited existing work studying the effect of the ethoxy groups on the phase formation and structure. This is particularly important for the effect the structure has on the viscosity, an important consideration for commercial products. Dissipative particle dynamics is used to simulate the full phase diagram of SLES in water, including both micellar and lyotropic liquid crystal phases. Phase transitions occur at locations which are in good agreement with experimental data, and we find that these boundaries can shift as a result of varying the number of ethoxy groups. Varying the ethoxy groups has a significant effect on the micellar shape and crystalline spacing, with a reduction leading to more nonspherical micelles and decreased periodic spacing of the hexagonal and lamellar phases. Finally, while typical commercial products contain a distribution of ethoxy groups, computational work tends to focus on simulations containing a single chain length. We show that it is valid to use monodisperse simulations to infer behavior about solutions with a polydisperse chain length, based on its mean molecular length.

## 1 Introduction

Surfactant molecules are an important component in many cleaning and personal care products. When the concentration of surfactant molecules in solution is above a critical concentration, the molecules will self-assemble into phases, a behavior which is driven by their amphiphilic nature. The particular arrangement of molecules taken is dependent on conditions such as the temperature,^1^ concentration,^1−4^ and surfactant type.^3^ Phases include micellar, lamellar, bicontinuous cubic, and hexagonal structures.^1−4^ In general, phases formed by ionic surfactants tend to be mostly dependent on variation of concentration, while for many nonionic surfactants, temperature is the most important variable.^5^ In this work, we study the formation of micellar phases and mesophases by anionic surfactants, where the relationship between the phase behavior and concentration is of particular interest, due to the widely differing properties that different phases possess. The liquid crystal phases have large viscosities^3,6^ (particularly the hexagonal structures), complicating the manufacturing process of surfactant-containing products.

Alkyl ethoxysulfates (AES) are common anionic surfactants with the chemical formula CH_3_(CH_2_)_*x*_(OCH_2_CH_2_)_*n*_OSO_3_Na, where *n* is the number of ethoxy (EO) groups and *x* + 1 provides the number of carbon atoms in the alkyl tail. These surfactants can be found in many personal care products, where there is typically a distribution of *n* and *x*. A special case of AES is described by molecules with a fixed hydrocarbon chain length, *x* = 11, which is typically referred to as sodium lauryl ether sulfate (SLE_*n*_S). Although SLE_*n*_S is a common component of many commercial products, there is limited published research studying its phase behavior in pure systems. It is most common to find literature reporting surfactant mixtures,^3,6,7^ and experimentally, there is more research dedicated to studying systems with polydisperse *n*,^3,8^ as opposed to monodisperse systems dedicated to understanding the effect of varying the number of EO groups. This is, in part, due to the difficulty in manufacturing pure SLES containing a single degree of ethoxylation. Therefore, the effect of varying *n* on the phase diagram is not well understood.

An exception to the abovementioned case is the special case in which *n* = 0 and *x* = 11, that is, sodium dodecyl sulfate (SDS), for which there is significant amount of research dedicated to the study of low concentration solutions using both experimental^1,9^ and simulation techniques.^10−13^ However, molecules with *n* > 0 are less frequently modeled, as are systems of higher concentration since focus is normally on the micellar region of the phase diagram.^14−17^ Computational studies allow us to investigate these surfactant systems on the molecular level; however, the only existing computational studies that could be found of single-component SLES surfactants were two recent studies, both of which focus on exclusively micellar concentrations. These include a study by Panoukidou et al.*,*^10^ who investigate SLES in sodium chloride/water solutions using dissipative particle dynamics (DPD), and Peroukidis et al.,^16^ who study SLES solutions using molecular dynamics (MD) simulations. In both of these studies, authors investigate micellar solutions where the degree of ethoxylation *n* is varied in the range (*n* = 1–3); however, in this work, we aim to extend beyond the micellar region alone.

The method of DPD is used in this work for simulating anionic surfactant solutions of SLES in water. Computational methods allow us to analyze the structure of phases in detail, much more easily than would be possible using experimental methods. DPD is particularly suited to studying polymer^18^ and surfactant systems,^13,19,20^ due to its ability to access long time and length scales compared with MD methods. We present a study of the phase diagram of pure, monodisperse SLES solutions as a function of composition and ethoxylation *n*. A polydisperse case is also studied, which will be referred to in this work as AES. The distribution of *n* for this case is chosen to replicate the distribution found in a typical commercial product. In order to compliment the DPD simulations, a selection of experimental measurements are performed to establish the phase diagram of commercial AES, since this could not be found in the existing literature for the type of AES modeled in this study. This is achieved using polarized optical microscopy (POM) and rheological measurements. POM uses plane-polarized light to observe structures that are birefringent, such as hexagonal and lamellar liquid crystals. However, micellar and cubic phases are optically isotropic; therefore, in order to distinguish between these phases, one can use the fact that they have extremely different viscosities.

This work is unique in its approach to studying the whole range of concentrations across the phase diagram. Typically, research focuses on either micellar solutions^10,11,16^ or liquid crystal phases,^20^ but rarely both. However, investigation of the whole diagram allows us to investigate the behavior at phase transitions, which are more difficult to assess experimentally. In this study, we investigate aspects of the structure which can also be difficult to measure in experiments, such as the effect of ethoxy groups on the micellar shape. Measurements of micelles can often produce conflicting results, depending on the experimental technique used.^21−23^ For the lyotropic liquid phases, we calculate values for their periodicity, including the lamellar layer *d*-spacing and the inter-rod spacing for hexagonal phases, as a function of EO groups and composition. Experimentally, lyotropic liquid crystals show a dependence of their periodicity on surfactant concentration.^24−28^ The *d*-spacing and inter-rod spacing, however, are rarely calculated using DPD simulations, due to the requirement of large simulation boxes to obtain meaningful results. Therefore, our simulation boxes are larger than those typically used by other authors.^13,19^

Finally, as previously stated, most computational work focuses on the simulation of monodisperse surfactant systems consisting of a single chain length. However, commercial products will typically contain a distribution of ethoxy groups, leading to the question of whether modeling commercial surfactants as a monodisperse simulation, using their average value of ethoxy groups, reproduces the same behavior as a true polydisperse system would. Polydisperse simulations are usually hindered by the finite number of molecules in the simulation box. This is a further contributing factor, as to why we choose large simulation boxes for this study.

## 2 Theoretical and Experimental Methods

### 2.1 Dissipative Particle Dynamics

**Figure 1:**
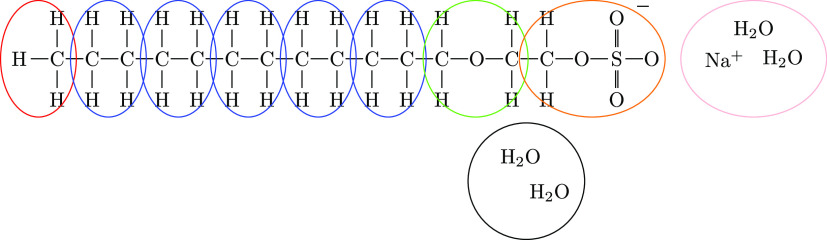
Coarse graining used where the number of [CH_2_OCH_2_] beads is varied.

The mesoscale simulation method of DPD was first introduced by Hoogerbrugge and Koelman^29^ and has been developed by many other contributors since then.^11,30−33^ The simulations performed in this work use simulation software DL_MESO.^34^ The DPD method coarse-grains groups of atoms into beads, and long chain molecules are modeled using a series of bonded beads. As these beads do not have hard sphere bounds, beads can overlap with each other. This contributes to the effect of a more rapid equilibration time, when compared with traditional molecular dynamics approaches. The coarse graining used is illustrated in Figure 1, and parameters for bead interactions are taken from Anderson et al.*,*^11^ who in their work apply their calculated parameters to modeling SDS molecules in the micellar region of the phase diagram. Water is coarse-grained such that one bead represents two water molecules. The sodium ion is modeled as partially hydrated, and one bead represents a sodium atom and two water molecules. The parameterization used in this work is based on calculating properties at 25 °C, and therefore, we focus entirely on behavior at room temperature.

The simulation boxes are three-dimensional, cubic domains, with edge length *L*. All simulations are performed at constant volume (NVT ensemble), and periodic boundary conditions (PBC) are applied to replicate the behavior of bulk fluid. The box is filled with an ensemble of beads, which are initialized with random placement. The force that acts on bead *i* can be written as
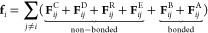
1where **F**_*ij*_ denotes the forces acting on bead *i* by bead *j*. The total force from nonbonded interactions is contributed from the conservative force **F**_*ij*_^C^, dissipative force **F**_*ij*_^D^, random force **F**_*ij*_^R^, and an electrostatic force **F**_*ij*_^E^. The forces **F**_*ij*_^C^, **F**_*ij*_^D^, and **F**_*ij*_^R^ are short-range and vanish beyond a defined cutoff *r*_C_. The repulsive conservative force is given by
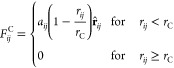
2where *a*_*ij*_ is the maximum repulsion between beads *i* and *j*, *r*_C_ is a specified cutoff radius, **r**_*ij*_ is the vector between beads *i* and *j*, **r**_*ij*_ = **r**_*i*_ – **r**_*j*_, *r*_*ij*_ = |**r**_*ij*_|, and . The conservative force provides beads with a chemical identity using constant *a*_*ij*_. The dissipative and random forces together form a thermostat, which keeps the mean temperature of the system constant. The dissipative force **F**_*ij*_^D^ and random force **F**_*ij*_^R^ are given by
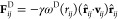
3
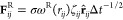
4where ω^D^ and ω^R^ are *r*_*ij*_-dependent weight functions that vanish for *r*_*ij*_ > *r*_C_, γ is a friction coefficient, *σ* is the noise amplitude, **v**_*ij*_ = **v**_*i*_ – **v**_*j*_, Δ*t* is the time step, and ζ_*ij*_(*t*) is a randomly fluctuating Gaussian variable with zero mean and unit variance. It was shown by Espanol and Warren^31^ that in order to satisfy the fluctuation–dissipation theorem, one of the weight functions, either ω^D^ or ω^R^, can be chosen arbitrarily, and this fixes the other weight function. The relationship between the two functions is shown to be
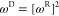
5and the relationship between the amplitudes is
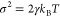
6where *k*_B_ is the Boltzmann constant and *T* is the temperature. In this work, we choose noise amplitude σ = 3, with the value for friction coefficient γ then defined by eq 6. A time step of Δ*t* = 0.01 is used, which has been shown to be adequate for accurate temperature control.^30^ The most commonly chosen function for ω^*D*^, and the one used in DL_MESO,^34^ is
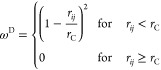
7Although other expressions can be chosen for ω^D^, the presented expression is often chosen, as it maintains the simplicity of the DPD method.

**F**_*ij*_^B^ and **F**_*ij*_^A^ are forces between beads which are directly connected (i.e., those that are chemically bonded). The force which holds bonded molecules together is represented by the spring harmonic potential
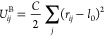
8where *C* is the spring constant and *l*_0_ is an unstretched bond length. Force **F**_*ij*_^B^ due to bond potential is then calculated using . A rigidity is introduced into the molecules, with the addition of a second harmonic potential
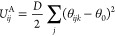
9where *D* is a constant, θ_0_ is an equilibrium bond angle, and θ_*ijk*_ is the angle between consecutive bonds. The force **F**_*ij*_^A^ is calculated similarly as mentioned above using . Values of *C* = 150, *D* = 5, and θ_0_ = 180° are used in this work. The equilibrium bond length *l*_0_ between a particular pair of beads is dependent on the number of “heavy atoms” involved (which are defined as C, O, and S in this work), using the formula
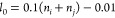
10where *n*_*i*_ and *n*_*j*_ are the number of heavy atoms in beads *i* and *j*, respectively. This assignment is based on a matching of the bond length in the hydrocarbon chain to that known experimentally, and more details about this can be found in Anderson et al*.*^35^

In molecular dynamics, atoms are typically treated as point charges for the purpose of evaluating the electrostatic force. However, the same treatment cannot be applied in DPD, as a result of the soft repulsions used between beads. Therefore, a typical approach is to use a smeared charge distribution. In order to model the electrostatic pair potential between charged beads, a Slater-type charge smearing^36^ is used, in which the Coulombic potential between two charged beads *i* and *j* is given by
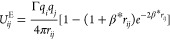
11where *q*_*i*_ and *q*_*j*_ are charges, Γ = *e*^2^/(*k*_B_*T*ε_0_ε_r_*r*_C_) is a dimensionless electrostatic coupling parameter, and β* = 0.929*r*_C_^–1^ is the tunable Slater parameter. Ewald summation methods^37−39^ can then be used to implement this calculation.

Typically, in DPD, the cutoff for each bead *R*_*ij*_^C^ is constant for all bead types and is usually set to be 1 DPD unit, that is, *R*_*ij*_^C^ ≡ *r*_C_ = 1. Differences in bead species are represented by choosing different values for *a*_*ij*_ in eq 2. However, in the Anderson et al.^11^ representation, both *a*_*ij*_ and *R*_*ij*_^C^ are varied. This allows different molar volumes to be captured by varying *R*_*ij*_^C^ between different bead species. A baseline cutoff distance is defined between solvent beads as *R*_WW_^C^ = *r*_C_ = 1, and other cutoffs are defined relative to this baseline. The exact choices of *a*_*ij*_ and *R*_*ij*_^C^ for different beads can be found in Anderson et al*.*^11^ The number density of water beads is set to ρ*r*_C_^3^ = 3. This choice of bead density is in line with other literature studies and is based on the work of Groot and Warren.^30^ The length scale of the system is defined by the cutoff distance for solvent beads, the energy on setting *k*_*B*_*T* = 1, and the mass on the DPD particle mass which is set to *m* = 1. A mapping of DPD units to real units is performed by matching the mass density in DPD to the mass density found experimentally for water at room temperature, producing a value *r*_C_ ≈ 5.65 Å. Throughout this work, results are presented in DPD units, and variables in units of length are presented in reduced units of *r*_C_. A time scale in real units can be obtained by making use of the relation . A summary of the DPD parameters required and their values, both in DPD and real units, can be found in Table 1.

**Table 1:** DPD Parameters Used in the Simulations and Conversion to Real Units

quantity	dimension	value (DPD units)	value (real units)
**length**	**L**	**1**	5.65 Å
**mass**	**M**	**1**	**5.98 × 10**^**–26**^ **kg**
**energy**	**E**	**1**	**4.11 × 10**^**–21**^ **J**
Δ*t*	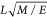	0.01	2.16 × 10^–14^ s
γ	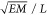	4.5	1.25 × 10^–13^ kg/s
ρ	L^–3^	3	1.66 × 10^28^ m^–3^
C	E/L^2^	150	1.93 J/m^2^
D	E	5	2.06 × 10^–20^ J

### 2.2 Simulation Setup and Box Size

DPD simulations are performed for a variety of concentrations in the range from 7 to 80% (note that all concentrations are presented as weight percentages). In this work, we choose to fix the length of the hydrocarbon chain to *x* = 11 (i.e., SLE_*n*_S surfactants) and vary *n* in order to investigate the effect that the number of ethoxy groups has on the phase diagram and phase structure. For each concentration, four monodisperse simulations are performed (for *n* = 0, 1, 2, and 3), as well as a case which has a distribution of *n*, corresponding to an AES product used by Procter & Gamble in the manufacture of commercial cleaning products. Initial simulations are performed in a box size of *L* = 20, in order to establish the type of phase generated for a particular value of concentration and number of ethoxy groups. Following this, we perform simulations in larger box sizes to study the phase structure. The increase in box size is found to have no influence on the type of phase produced, within the resolution of concentration used in this study.

**Figure 2:**
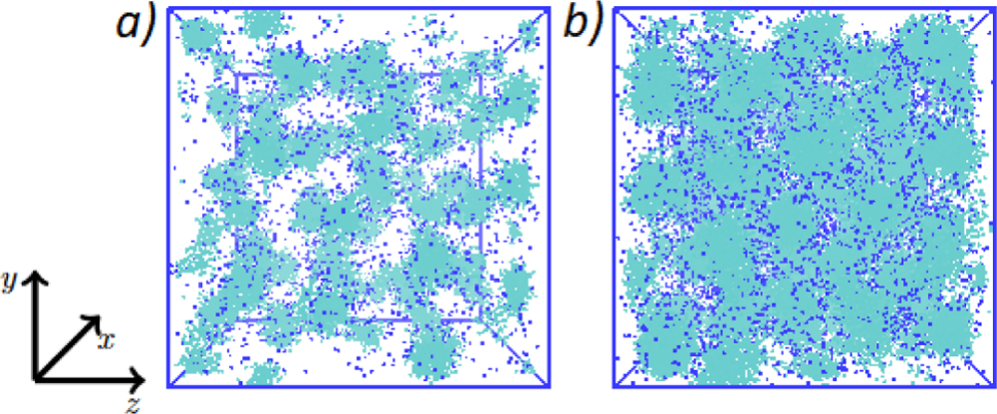
Micelles formed by surfactants with *n* = 1 ethoxy groups at two different concentrations: (a) 7 and (b) 20%. Beads are colored according to their type: light blue (surfactant chain) and dark blue (sodium ions). Water molecules are not shown. Figure created using VMD.^40^

For concentrations 7, 10, 20, and 30%, which are expected to produce micellar solutions, a box size of *L* = 50 is used. Although this is significantly larger than box sizes which are typically used to study the micellar phase,^10,11,13^ a large simulation box is chosen to ensure that a distribution of micelle sizes is produced. This, of course, has the effect of requiring a longer equilibration time. In order to determine that the micellar solution is fully equilibrated, the mean aggregation number of the system *N*_agg_ is monitored as a function of iteration. The point of equilibration is determined as the point when no further change in *N*_agg_ is observed with further iterations. We define this as the point at which block averages (where a block is 5000 DPD time units) of *N*_agg_ becomes constant (within reasonable fluctuation). In order to calculate *N*_agg_, individual micelles need to be identified. Clusters are identified by defining a cutoff distance, and molecules that are closer than that distance are said to be in contact with each other and form an aggregate. Only the hydrophobic tail of the molecules is used in this calculation, as they are expected to make up the hydrophobic core of the micelle. The cutoff distance used in all of the calculations in this work is 1 DPD unit. A minimum of 2.5 × 10^7^ iterations were found to be sufficient for equilibration, requiring approximately 52,000 CPU hours (for parallel simulations, split across 27 processors). An example of final equilibrated micellar phases is illustrated in Figure 2.

For the liquid crystal phases, the periodicity of the structure must be taken into account when choosing the box size. For the lamellar bilayers, this periodicity is characterized by the *d*-spacing. The bilayers form at an orientation, relative to the simulation box surfaces, such that the lamellar layers have a *d*-spacing which minimizes the potential energy of the system. However, due to the fact that the simulation box has finite size and PBCs, there are constraints on the *d*-spacings at which bilayers can form. The *d*-spacing layers must satisfy
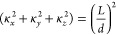
12where κ_*i*_ denotes integers related to the number of layers that have formed in dimension *i*, where *i* = *x*, *y*, and *z*. Therefore, increasing *L* increases the number of “available” *d*-spacings for a particular system. Box size *L* = 40 is chosen for studying lamellar phases in this work. This size is chosen as it allows for a large number of potential *d*-spacings, balanced against the increased computational cost of a larger box size.

**Figure 3:**
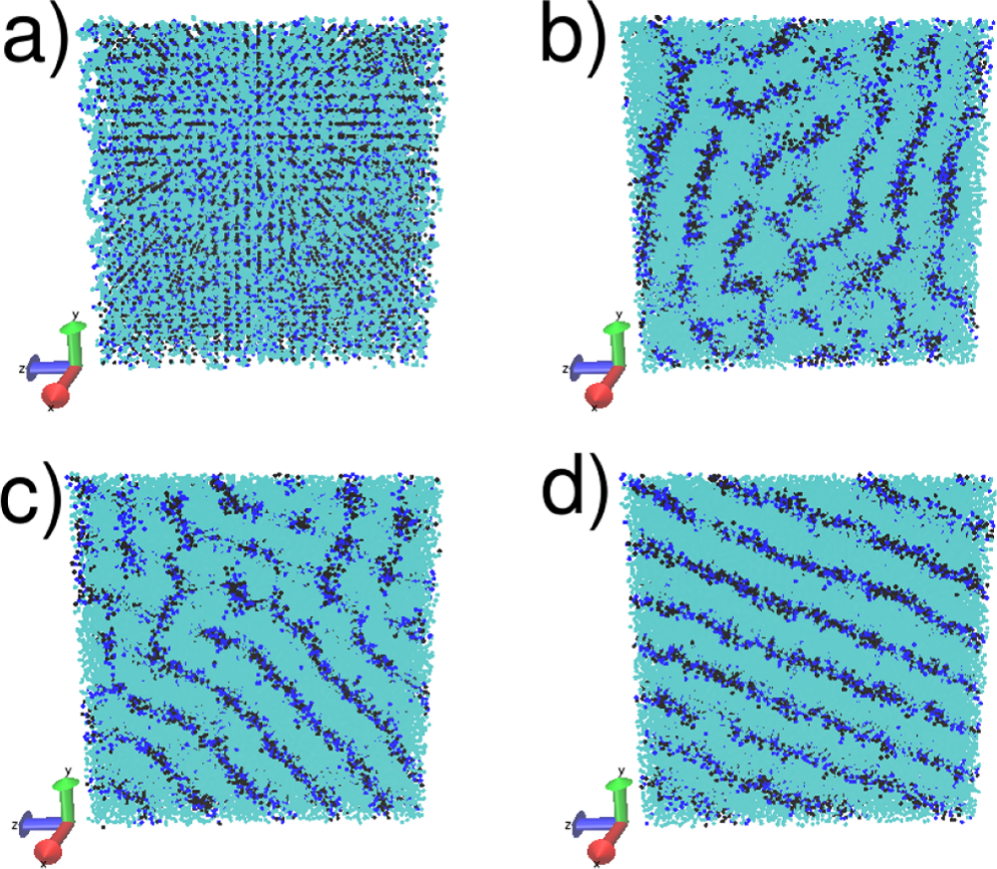
Equilibration of a case in which the number of ethoxy beads *n* = 0 and concentration *c* = 80%, which results in lamellar layers, is shown at different iterations *I*. (a) *I* = 0; (b) *I* = 2 × 10^5^; (c) *I* = 1.4 × 10^6^; (d) *I* = 2.2 × 10^6^. Beads are colored according to their type: light blue (surfactant chain), dark blue (sodium ions), and black (water). Figure created using VMD.^40^

The lamellar structure forms over a relatively smaller number of iterations, when compared with the micellar and hexagonal phases. Visual inspection is used in order to confirm that the equilibrium structure has been achieved, that is, when there are clear, parallel layers of alternating water and surfactant molecules. The hydrocarbon tails of the surfactants are orientated toward the center of the bilayers, and the Na^+^ ions reside in the parallel water layers. The lamellar systems of higher concentration generally take fewer iterations to reach an equilibrium state than those with lower concentrations. For example, for the system containing surfactant molecules with *n* = 1, the *c* = 80% case requires 7.2 × 10^6^ iterations to equilibrate, which is equivalent to approximately 6,000 CPU hours, while for the same surfactant molecule, a system consisting of *c* = 60% takes 1.3 × 10^7^ iterations, equivalent to approximately 10,000 CPU hours. Equilibration time is also reduced when there are fewer ethoxy units *n* in the surfactant chain, for example, when *n* = 0, the *c* = 80% case takes only 2.2 × 10^6^ iterations to equilibrate. An example of the equilibration process of a lamellar phase from an initial configuration is shown in Figure 3.

**Figure 4:**
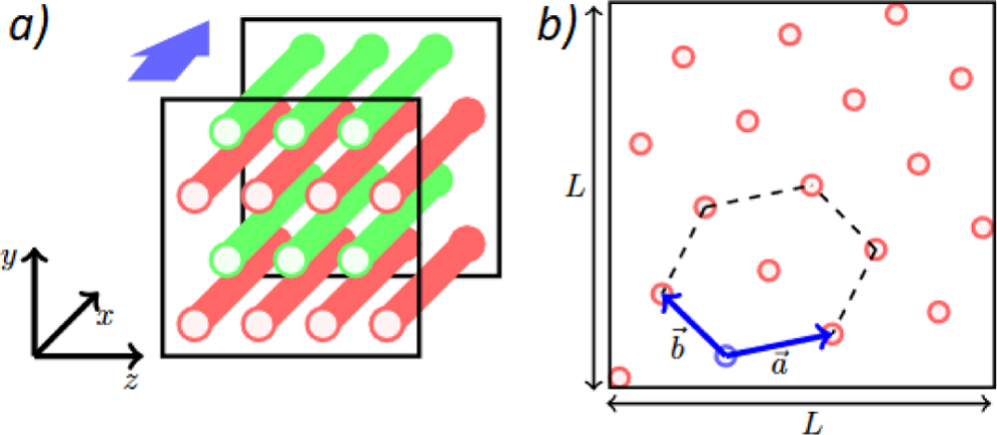
Application of shear force causes hexagonal phase alignment in the *x*-direction (a), so that the inter-rod spacing is optimized by orientating in the *y*–*z* plane (b). Direction of flow is indicated by the thick arrow (a).

The inter-rod spacing *r*_S_ of the hexagonal phase is also restricted due to the application of PBCs. In this work, all hexagonal phases form with rods parallel to the *x*-axis, as illustrated in Figure 4. This is due to the application of shear which causes alignment in one dimension. Therefore, the hexagonal phase must optimize its inter-rod spacing by orientating within the *y*–*z* plane only. It can be shown that the unit cell must satisfy
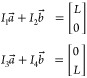
13where *I*_*i*_ denoted integers, and lattice vectors are defined in Figure 4. The hexagonal phase was observed to take a long time to form under equilibrium conditions, relative to the micellar and lamellar phases. This behavior has also been found in DPD simulations for other surfactant systems.^41,42^ The reason for the application of shear is that, under equilibrium simulation conditions, a number of solutions in the concentration range 40–60% have the appearance of worm-like micellar phases. When shear force is applied, these cases can form a structured hexagonal phase. Furthermore, upon removal of that shear, the hexagonal structure is maintained. This is the mechanism for generating the hexagonal phases presented in the DPD phase diagram. Under equilibrium conditions (no shear), we tried up to 10^8^ iterations and find that no cases result in fully equilibrated hexagonal phases. In contrast, we found that the typical number of iterations to form hexagonal phases with shear was just 3 × 10^6^ iterations.

A perfect hexagonal lattice requires that . However, in order to satisfy eq 13, the hexagonal lattice is found to stretch/obscure in order to satisfy the boundary conditions. For a perfect hexagonal lattice, the distance to the nearest six neighbors is the same, which defines the inter-rod spacing value as . However, for the lattice described in Figure 4, there can be up to three independent distances for the six nearest neighbors to a particular rod, described by the length of vectors |*a⃗*|, |*b⃗*|, and . Therefore, an average inter-rod spacing *r*_S_ is calculated as an average of these three nearest-neighbor distances. Experimentally, the inter-rod spacing and lamellar *d*-spacing values are typically obtained by small-angle X-ray scattering,^3,43,44^ and the experimental data are fit under the assumption that . This means that while the inter-rod spacing value presented in this work is, strictly speaking, not equivalent to that which is calculated experimentally, it should provide a value which is similar. Therefore, any variation in *r*_S_, which results from changing the concentration or the number of ethoxy groups, should be observable.

### 2.3 Experimental Phase Diagram

The experimental phase diagram of AES is presented in this work, in order to compare with DPD results. The AES paste used in these experiments is provided by Procter & Gamble, with the distribution of chain lengths and ethoxylation presented in Table 2. However, in our DPD work, due to the finite number of molecules that can be implemented in a simulation, the distribution is simplified to only contain molecules with chain length *x* = 11 and *n* = 0–3. This means that the average value of ethoxylation for AES differs slightly between the AES paste (*n* ≈ 1) and AES in DPD (*n* ≈ 0.76).

**Table 2:** Distribution of Hydrocarbon Chain Length *x* and Ethoxy Groups *n* in AES Paste Compared with the Distribution in the Simplified DPD Representation

*x*	Exp (%)	DPD (%)
11	68	100
12	0	0
13	26	0
14	0	0
15	6	0

*n*	Exp (%)	DPD (%)
0	49	52.7
1	24	25.8
2	13	14.0
3	7	7.5
4	4	0
5	2	0
6	1	0
7	0	0

The phase diagram of AES at room temperature is established using POM and rheological measurements. Homogeneous samples were prepared by mixing AES paste with deionized water to create the desired concentration and leaving the sample to stand at room temperature. Rheology measurements were performed at 25 °C using an Anton Paar Physica MCR301 rheometer. Two different geometries are used depending on the observed qualitative viscosity. Low viscosity samples in the range 7–20% are measured using a concentric cylinder geometry (27 mm-diameter cylinder and gap size 1 mm), while all samples above this concentration are measured using a cone and plate geometry (diameter of the upper plate 75 mm and 1° angle). For each sample, a variety of shear rates are trialed, determining the relationship between the viscosity and applied shear rate. Measurements were performed in the largest shear-rate-range possible. The lower bound on shear rate is dictated by the minimum torque accessible (0.1 μNm). For samples measured in the concentric cylinder geometry, at higher shear rates, there can be an influence of secondary flows, and Couette flow can no longer be assumed, placing an upper bound on what can be measured. For each concentration measured, we begin at low shear rates, and a logarithmic stepwise ramp method was used to gradually increase the shear rate.

## 3 Results and Discussion

### 3.1 AES Experimental Phase Diagram

**Figure 5:**
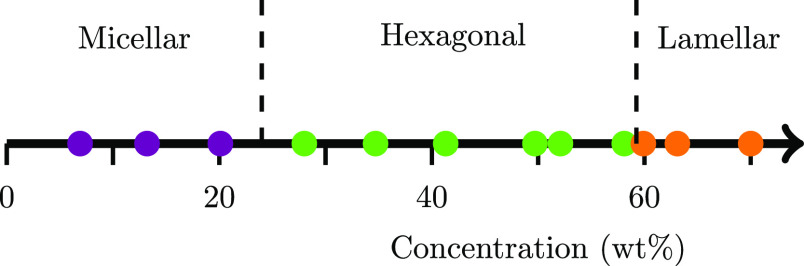
Phase boundaries of AES solutions at room temperature, identified by POM imaging and rheological measurements.

Micellar phases are easily identifiable from their rheological behavior, due to the fact that liquid crystals have significantly larger viscosities. Concentrations in the range 7–20% have low viscosity and display a Newtonian relationship with shear rate when measured in the range γ̇ = 1–60 s^–1^. In this concentration range, the solutions exhibit no textures when viewed by POM, confirming the presence of the micellar phase. Samples in the concentration range 28–70% display significantly larger viscosity values and exhibit shear thinning behavior under shear rates in the range γ̇ = 0.001–0.1 s^–1^. All samples in this concentration range display textures when viewed using POM, indicating that the solutions possess a hexagonal or lamellar structure. A summary of the viscosity measurements as a function of composition is shown in Table 3. Note that an attempt was made to measure the viscosity of a sample with a concentration of 58.6%; however, the viscosity was too large to be accurately measured at lower shear rates and is therefore omitted from the table. However, a large viscosity implies the presence of a hexagonal phase at this concentration, as transition to a lamellar structure would be thought to be identifiable from a decrease in viscosity. Furthermore, if the 58.6% sample had a cubic structure, this would be identifiable from a lack of textures under POM imaging. A summary of the identified phases as a function of concentration is shown in Figure 5. Further details can be found in the Supporting Information.

**Table 3:** Viscosity Range of AES Solutions for Different Phases^a^

phase type	concentration range **(wt %)**	viscosity range **(Pa**·**s)**
micellar	6.9–20.1	(1.6 × 10^–3^)–(9.2 × 10^–3^)
hexagonal	28.0–52.1	(1.5 × 10^3^)–(1.7 × 10^4^)
lamellar	59.9–70	(6.5 × 10^2^)–(1.2 × 10^4^)

a Samples were measured in the range γ̇ = 0.001–0.1 s^–1^.

In the micellar range of concentrations (7–20%), the viscosity was observed to increase with increasing concentration, which is consistent with what is reported for similar systems.^45,46^Figure 5 reports a concentration of 28% as having a hexagonal structure, due to its textures under POM and increased viscosity relative to the micellar phase. However, it is worth noting that the rheology at this concentration is markedly different to the hexagonal solutions at higher concentrations. In particular, the measured viscosity in the low shear rate region is significantly lower than it is for subsequent samples at 35% ≤ *c*. This may be a result of a mixed micellar/hexagonal phase under equilibrium conditions and/or shear-induced phase changes for concentrations on the micellar-hexagonal boundary. Indeed, it has been observed that worm-like micelles can thicken under shear, due to an induced structure.^47^

The phase transition order micellar → hexagonal → lamellar which is observed for AES is similar to the reported phase diagrams of pure SDS^1^ and SLE_3_S,^2^ which is to be expected based on their similar molecular structure. When the number of ethoxy groups is large (e.g., for SLE_3_S^2^), there can sometimes be an additional bicontinuous cubic phase which is not observed for shorter molecules (e.g., for SDS) between the hexagonal and lamellar phases. This, however, is not observed for AES.

### 3.2 Simulation Results

#### 3.2.1 Phase Diagram

**Figure 6:**
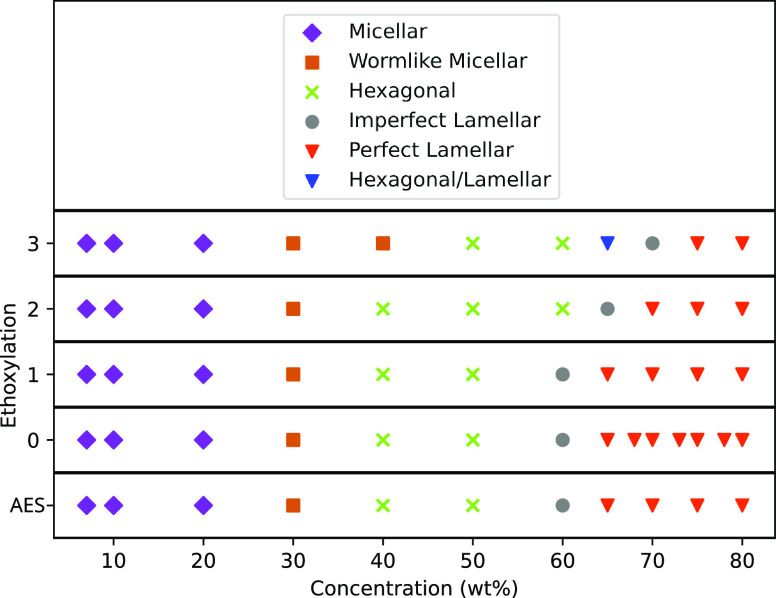
Phase diagram at room temperature, as determined via DPD, for varying values of ethoxylation *n*.

As previously discussed, in order to encourage hexagonal phase formation, a shear force is applied to the simulation box in the *x*-direction. This means that the hexagonal phase can be induced in a significantly reduced simulation time. As a result, the reported phase diagram is produced from a mixture of equilibrium and shear-induced mesophases. The determined phase behavior is shown in Figure 6, as a function of concentration and ethoxy groups *n*. There is a small shift in the location of the phase boundaries as the value of *n* increases. Five main phases are produced, including a micellar region, a worm-like micellar phase, a hexagonal phase, and two lamellar phases (classified as “perfect” and “imperfect”). There is also a sixth mesophase labeled “hexagonal/lamellar”. In this case, the phase displayed a structure that was a hybrid of the hexagonal and lamellar phases, displaying characteristics of both.

The phase diagram for AES is largely consistent with the experimental diagram in Figure 5. The micellar/hexagonal phase boundary is experimentally determined to be in the range 20–28%, while in simulation, it is located in the higher concentration range 30–40%. DPD, however, allows us to probe the phase boundaries more carefully and analyze potential ambiguity in the experimental phase boundaries previously discussed.

For all degrees of ethoxylation *n* trialed, the lamellar phase is produced at high concentrations. However, the lamellar phase is not usually found experimentally for SDS at room temperature,^1,9^ although it has been reported by other authors modeling SDS using DPD.^13^ At room temperature, SDS solutions at high concentration are experimentally reported as having an inhomogeneous concentration distribution.^1^ In this region, there is a phase separation into mixtures of crystalline SDS and low-concentration phases. The lamellar phase produced in DPD may be due to the fact that this behavior would be hard to reproduce in DPD, due to the relatively small box size (relative to the length scales in a nonuniform solution).

For all values of *n*, there is a transition directly from the hexagonal phase to the lamellar phase. Experimentally, a cubic phase for SLE_3_S has been observed between these two mesophases in the concentration range 62–67%.^2^ However, since we encourage the hexagonal phase to form in DPD via the application of shear, this may have the consequence of causing a phase transition from a cubic phase into a hexagonal structure, explaining why no cubic phases are observed.

The structure of the identified phases will be discussed in the remainder of this paper.

#### 3.2.2 Micellar Phase

The effect of varying the concentration and ethoxylation *n* on the final *N*_agg_ achieved is shown in Table 4, with *N*_agg_ increasing approximately linearly with concentration. Interestingly, aggregation number *N*_agg_ is found to be largely independent of *n*, which has also been observed experimentally.^23^ The increase in *N*_agg_ for higher concentrations is what is expected from experimental measurements.^48,49^ However, in general, the aggregation number is under predicted, although the gap between experiment and DPD narrows with increasing concentration. For example, the aggregation numbers for SDS micelles at concentrations 10 and 20% are compared with experimental values in Table 5, showing that the underprediction for a concentration of 20% is significantly lower compared with 10% solutions.

**Table 4:** Final Mean Aggregation Number *N*_agg_ for Micellar Solutions of Varying Concentration *c* and Degree of Ethoxylation *n*^a^

	*c* = 7%	*c* = 10%	*c* = 20%
*n* = 0	44 ± 12	54 ± 10	80 ± 19
*n* = 1	41 ± 13	48 ± 14	94 ± 24
*n* = 2	41 ± 10	52 ± 10	94 ± 25
*n* = 3	39 ± 11	51 ± 13	83 ± 22
AES	46 ± 12	57 ± 15	88 ± 17

a Errors represent the standard deviation over the sampling period.

**Table 5:** Aggregation Numbers *N*_agg_ for SDS Micelles at Room Temperature with Varying Concentration *c*^a^

*c* (wt %)	*N*_agg_^Exp^	*N*_agg_^DPD^	*N*_agg_^DPD^/*N*_agg_^Exp^
10	104	54	0.52
20	112	80	0.71

a Experimental values obtained from small-angle neutron scattering measurements performed by Hammouda.^49^

Although the average aggregation number is lower than that found experimentally, there are a sufficient number of micelles in the system that their shape can be analyzed as a function of size. The shape of micelles is analyzed using the radius of gyration *R*_G_, which is calculated using the relation
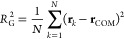
14where **r**_*k*_ is the position of a particle in a micelle consisting of *N* particles and **r**_COM_ is the center-of-mass for the micelle: . If one assumes spherical micelles with constant density, there is a linear relationship between micelle radius *R*_S_ and the radius of gyration
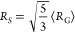
15which can be used to compare with experimentally determined values for micelle size. When micelles begin to elongate, this linear relationship no longer holds. However, *R*_G_ can still be used to characterize the shape of micelles. Using the fact that the volume of the hydrophobic core is proportional to *N* (i.e., the density is independent of the aggregation number), then spherical micelles will follow the relation *R*_G_ ∝ *N*^1/3^. Therefore, *R*_G_^3^/*N* is independent of *N* for spherical micelles and can be used to identify the value of *N* at which the micelles become nonspherical.^11^ Note that throughout this work, we use *N* to denote an aggregation number for a particular micelle, while *N*_agg_ refers to the mean aggregation number.

**Figure 7:**
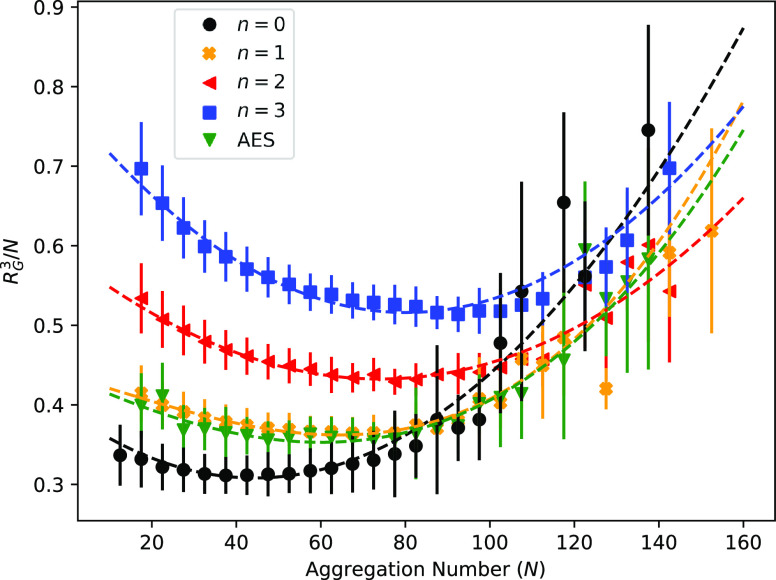
*R*_G_^3^/*N* against aggregation number *N* for a variety of solutions with varying ethoxylation *n* values. In this figure, the results from all three concentrations simulated (7, 10, and 20%) are combined. The aggregation number *N* is binned into bins of size 5, and error bars represent the standard deviation.

Radius of gyration *R*_G_ is calculated for concentrations *c* ≤ 20%. The relationship between *R*_G_ and *N* in a particular micellar solution is found to be strongly dependent on the ethoxylation *n*; however, the solution concentration has minimal impact. Therefore, the results from simulations with varying concentration are combined for the purpose of studying micellar shape. Figure 7 shows the comparison of the relationship between *R*_G_^3^/*N* and *N*, showing that the value of *N* which produces spherical micelles varies with ethoxylation *n*.

**Figure 8:**
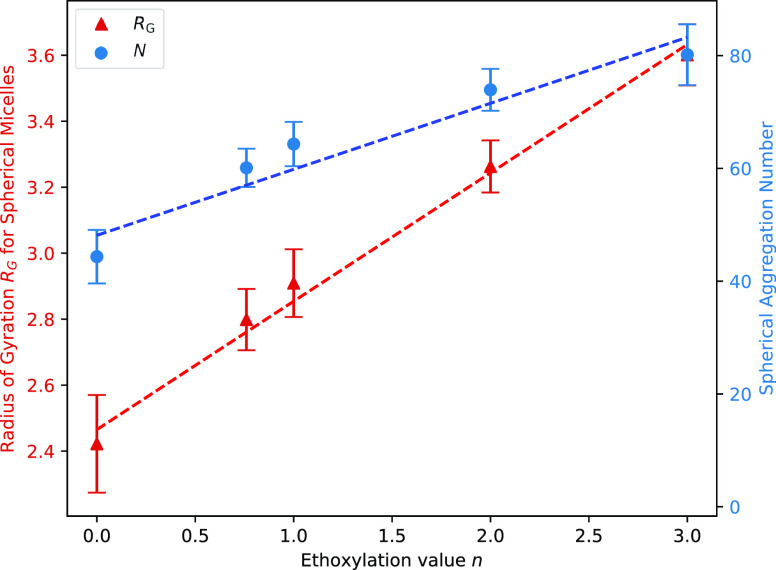
Radius of gyration *R*_G_ (DPD units) and mean aggregation number are calculated for micelles which are deemed to be approximately spherical, based on the value of *R*_G_^3^/*N*. Plotted is the variation of these two quantities with varying number of ethoxy groups *n*. Error bars shown represent the standard deviation.

It is observed that the range in *N* for which *R*_G_^3^ ∝ *N* holds is relatively narrow, and at low and high values of *N*, the relationship deviates, suggesting a nonspherical nature. It is interpreted that the deviations from linearity at low aggregation numbers are a result of prolate shape, while deviations at high aggregation numbers show a transition from spherical to oblate shape. For smaller values of *n*, the micelles become nonspherical more rapidly with increasing aggregation number. The location of the curve minima can be used to calculate the size of spherical micelles. Figure 8 shows the relationship between spherical micelle aggregation number and *n*, as well as the radius of gyration for these spherical micelles. Also plotted are the values for micelles containing a distribution of *n*, located at *n* = 0.76, representing AES. The linear relationship between *R*_G_ and *n* indicates that the spherical micelle radius is proportional to the molecular length, which logically makes sense for a micelle with diameter *D* ≈ 2*R*_S_. The aggregation number at which spherical micelles form increases with increasing *n*. This implies that decreasing the number of ethoxy groups leads to an increase in more rod-like micelles, as opposed to more spherical micelles for higher *n* values. This behavior has been observed experimentally for similar surfactants.^50^

The values of *R*_G_ and aggregation number calculated for AES solutions (with an average value of *n* = 0.76) fit in reasonably well with the trends calculated from the monodisperse cases. It is also worth noting that the distribution of *n* in a given micelle was independent of its size and was consistent between micelles. This means that there was no observed preference for surfactants to form micelles with molecules of a similar degree of ethoxylation. In the AES simulations, micelles formed from molecules of different lengths, in the same ratios as those present overall.

We find that as the concentration grows, we find more nonspherical micelles, eventually transitioning into entirely worm-like micelles (30% concentration). It was highlighted in Section 3.1 that the viscosity grows for micellar solutions as a function of concentration. Changes to the solution viscosity are primarily influenced by changes in the shape of micellar aggregates, the number of micelles formed, and/or due to micellar interactions.^45,46,51,52^ It is often suggested that strong repulsive inter-micellar interactions play a large role in the measured increase in viscosity.^45,46,52^ We observe that the increase in viscosity is likely to be a result of a combination of shape effects, as well as inter-micellar interactions. The gradual transition that we find between the micellar and hexagonal phase is in agreement with what is observed in a recent MD study^53^ for cetyltrimethyl ammonium chloride surfactants.

In summary, we find that for the simulations conducted in the range 7–20%, the bulk of the micelles formed have relatively spherical shape. Varying the number of ethoxy groups *n* leads us to conclude that there is an “ideal” micellar aggregation number for which the micelles are the most spherical. Decreasing *n* shifts this ideal value toward smaller aggregation numbers, leading to more nonspherical micelles in solution. Upon the increase from concentration 20 to 30%, the micelles become significantly worm-like and interwoven, such that the micelles become more difficult to distinguish, and calculating the radius of gyration becomes difficult. These simulations indicate a relatively smooth transition from the micellar to the hexagonal phase, due to a gradual growth of spherical to worm-like micelles, upon increasing concentration. When these worm-like micelles reach sufficient length, they can form infinite rods which stack to form the hexagonal phase, which will be discussed in the following sections.

#### 3.2.3 Lamellar Phase

The lamellar phases are categorized into two different cases: “perfect” and “imperfect”. When the concentration is high, the surfactants typically organize into well-defined layers of alternating water and surfactant molecules. Separate water layers do not connect once the phase has formed, and similarly, alternating surfactant layers also remain disconnected from each other (which describes the “perfect” case). However, for concentrations near the hexagonal-lamellar phase boundary, the lamellar structures can have bridges which spontaneously form and disconnect between the alternating water layers (describing the “imperfect” case). These bridges are shown to persist in the simulation, although still in dynamic motion, even with further iterations.

**Figure 9:**
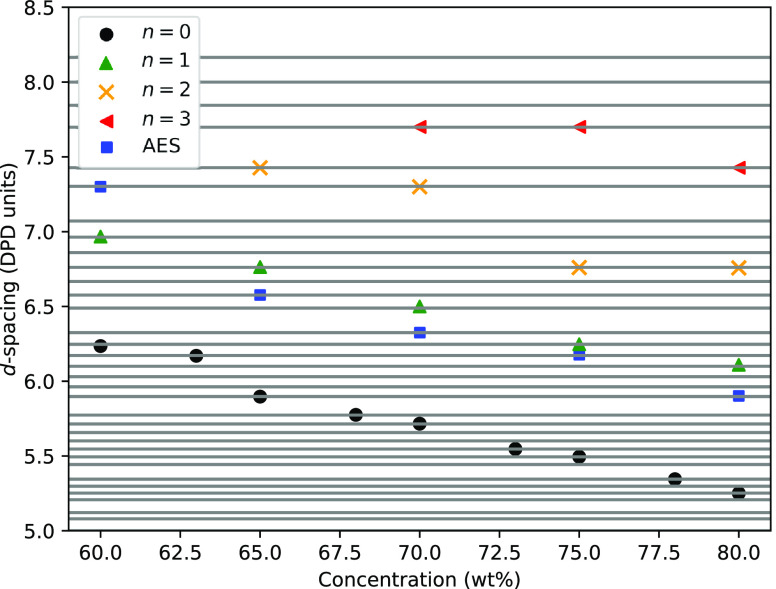
*d*-spacing values for the lamellar phase, for varying concentration and ethoxylation *n*, in a box of size *L* = 40. The horizontal lines in gray represent the available *d*-spacings for a box of size *L* = 40, as calculated using eq 12.

**Figure 10:**
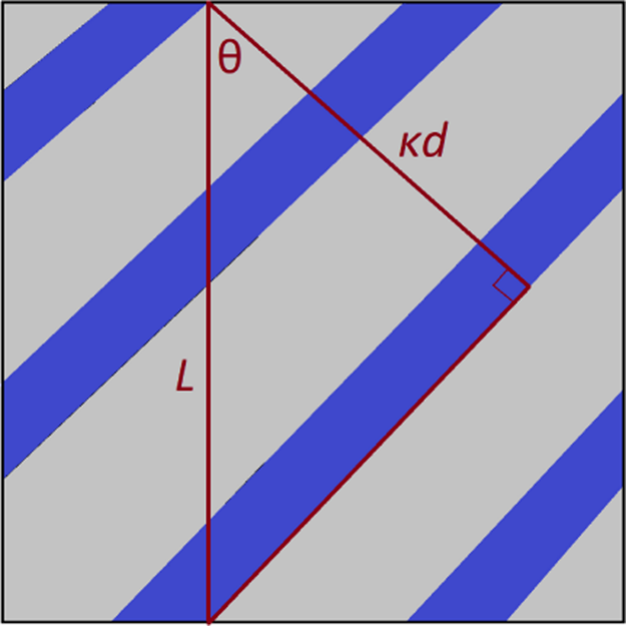
Two-dimensional illustration of lamellar layers in the simulation box. A triangle illustrates the constraint that the PBCs impose, leading to expression eq 16.

Figure 9 shows the effect of concentration and ethoxy groups on periodicity. Note that although results are presented in DPD units, a conversion to real units can be performed by multiplying by *r*_C_ = 5.65 Å (see Table 1). Also shown are the available values of the *d*-spacing for a box of size *L* = 40, provided by eq 12. The *d*-spacing presented in Figure 9 is calculated by making use of the director vector of the surfactant molecules. Defining angle *θ* as the polar angle to this unit vector, bilayers must form in order to satisfy the relationship *L* cos *θ* = *κd*, where *d* is the *d*-spacing and *κ* is the number of bilayers formed. This relationship is illustrated in Figure 10. This then leads to an efficient method of calculating the *d*-spacing for a lamellar layer system, as simply
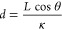
16The angle θ is found to be able to be calculated with a high degree of precision, due to the large number of molecules in the simulation box, making this a very accurate method for determining a value for *d*. This method is found to be accurate for both the “perfect” and “imperfect” simulation cases. Although there is more noise in the director for the imperfect simulation cases (due to the spontaneous formation and breaking of bridges), the calculation is still accurate due to the large number of configurations used.

Figure 9 shows that the layer separation increases with increasing *n* and decreases with increasing concentration. This trend with concentration is consistent with what is found experimentally for most types of surfactants.^25,26,44^ It is interesting to note that it is not simply the magnitude of the *d*-spacing calculated which determines whether the lamellar phase is classified as “perfect” or “imperfect”. Experimentally, values for *d*-spacing and inter-rod spacing are most often reported for systems with a mixture of surfactant types^54^ (i.e., a solution containing two or more different types of surfactants), so experimental data for pure systems are limited. A comparison of two DPD systems with experimental values is shown in Table 6. Although there is excellent agreement for the solution case with *n* = 3, there is more of a discrepancy between the results for AES. Since, in order to model AES using a finite number of molecules, the distribution for AES was simplified (see Table 2), most of the long molecular chains were removed from the distribution. This simplification is likely to contribute to an underprediction for the *d*-spacing in this case. It would be of interest in future work to also consider the effect of varying the length of the alkyl chain, as well as inclusion of longer chains into the simulations modeling AES.

**Table 6:** Comparison of Experimental and Simulated d-Spacings at *c* = 70% (AES) and *c* = 72% (SLE_3_S)^a^

type	experiment (nm)	DPD (nm)
AES	4.05^55^	3.57
SLE_3_S	4.39^2^	4.35

a Note that although a calculation was not performed for *n* = 3 at *c* = 72%, values for 70 and 75% are identical in simulations.

There is only a small amount of difference between the spacing formed by the *n* = 1 and AES simulation cases. The AES distribution case has an average value of *n* = 0.76 in the simulations, which explains why the values for AES are slightly below the ones for when *n* = 1. The fact that the simulation is a distribution of *n* seems to have relatively little impact on the *d*-spacing formed, beyond being impacted by the average value of *n* (i.e., there is no positional preference for molecules depending on their value of *n*). An interesting consideration is whether the *d*-spacing for the AES case can be calculated using the *d*-spacing from the monodisperse simulations (with *n* = 0 and *n* = 1). Therefore, we calculate values
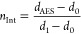
17for each value of concentration. In this equation, *d*_0_, *d*_1_, and *d*_AES_ are the *d*-spacings for the *n* = 0, *n* = 1, and AES cases, respectively. For concentrations 65–80%, we find that this takes an average value of *n*_Int_ = 0.81 ± 0.03 (where *c* = 60% is excluded as it is assumed to be an anomalous value). Comparing this with the average ethoxylation of AES as *n* = 0.76 implies that the *d*-spacing for the polydisperse lamellar phase can be reasonably interpolated from the monodisperse lamellar calculations.

**Figure 11:**
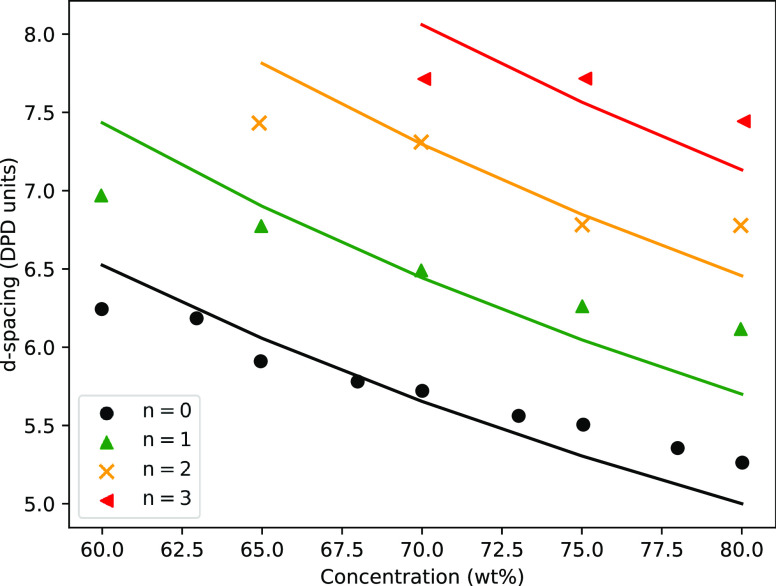
*d*-spacing values for the lamellar phase, for varying concentration and ethoxylation *n*, in a box of size *L* = 40. Fits are applied using eq 18.

The decrease in *d*-spacing with increasing concentration can partially be explained by the change in the ratio of surfactants to water beads in the system. We assume that the thickness of the surfactant layer *d*_s_ can be estimated as *d*_s_ = *N*_s_*V*_s_/*A*, where *V*_s_ is the volume of a single surfactant molecule, *N*_s_ is the number of surfactants in the layer, and *A* is the area of the interface between water and surfactant molecules. The *d*-spacing (combined water and surfactant thickness) can similarly be calculated as *d* = (*N*_s_*V*_s_ + *N*_I_*V*_I_ + *N*_w_*V*_w_)/*A*, where *V*_I_ and *V*_w_ are the ion and water bead volumes, respectively, and *N*_I_ and *N*_w_ are the number of ions and water beads in a single layer, respectively. The *d*-spacing can therefore be estimated as
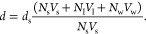
18The volume of a bead is calculated using its cutoff value for the radius, and the volume of a surfactant is calculated as the sum of these volumes. If we assume that for a given value of *n*, *d*_s_ is independent of concentration, this allows us to fit eq 18 to the data presented in Figure 9. The results of this fitting are presented in Figure 11, where the value of *d*_s_ is a fitted variable and varies with *n*. We observe that the decrease in *d*-spacing can partially be explained by the changes in the volumes of surfactant molecules and water beads present for a given concentration. The lack of a perfect fit implies that there is also a moderate change in *d*_s_ with concentration; however, fitting with variable *d*_s_ was not performed.

In summary, the lamellar bilayers can vary their orientation in order to form an optimal *d*-spacing value, minimizing the potential energy of the system. The *d*-spacing is found to vary as a function of both the system concentration and the number of ethoxy groups in the surfactant chain, and the *d*-spacing values calculated for the lamellar phase show good agreement with the limited experimental data available. There is also evidence that for polydisperse simulations, the *d*-spacing can be predicted from an average degree of ethoxylation (similar to the micellar phase previously discussed). In the following section, we study the hexagonal phase in more detail, including a quantification of the periodicity, similar to the *d*-spacing calculated for the lamellar phase.

#### 3.2.4 Hexagonal Phase

It is suspected that the cause of poor equilibration for hexagonal phases without the application of shear is due to the systems becoming trapped in local minima configurations. Worm-like micelles form across the PBCs and become interwoven. There are likely to be large energy barriers associated with breaking apart these worm-like micelles and the reforming of straight rods across the boundary.

Of course, one must consider if the application of shear transforms a solution which forms a lamellar phase under equilibrium conditions into a hexagonal phase upon shearing, in order to confirm that this approach would not misidentify the hexagonal/lamellar phase boundary. This is investigated by applying shear to lamellar phases with concentrations on the edge of the boundary with the hexagonal phase. It is confirmed that the lamellar phase forms under both equilibrium and sheared conditions, and no shear-induced phase change is observed. Likewise, for the phases identified as “worm-like micellar” in Figure 6, no hexagonal phase is observed under shear. In these cases, the worm-like micelles typically align in the direction of shear flow, although the concentration is not high enough for the formation of infinite rods, and any alignment induced is lost when the shear is removed.

Application of a shear force has the effect of orientating the hexagonal phase such that the rods are in line with the direction of shear flow. This was expected from experimental observations of shear-induced phase alignment in hexagonal structures.^56−58^ The shear flow is induced in DPD using Lees-Edwards boundary conditions,^59^ with the resulting alignment of rods previously shown in Figure 4. The rods are still free to position themselves within the *y*–*z* plane, in order to achieve an optimal inter-rod spacing.

**Figure 12:**
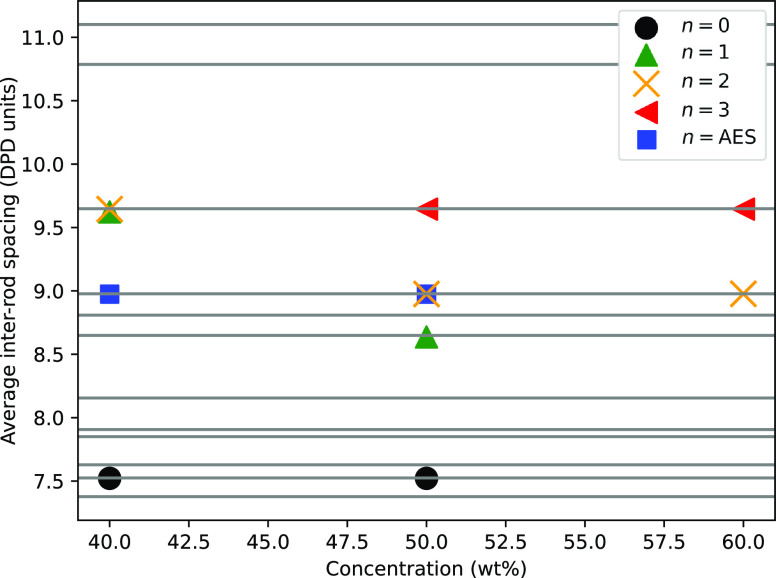
Average inter-rod spacing value formed in hexagonal phases at different concentrations and values of ethoxylation *n* in the surfactant chain. The horizontal lines are the available spacings, as obtained by the method described in the text.

The relationship between the average inter-rod spacing *r*_S_ as a function of ethoxy groups *n* and concentration is presented in Figure 12. There is a clear increase in the inter-rod spacing with increasing *n*. There is also evidence of a decreasing inter-rod spacing, as the surfactant concentration increases, which is in agreement with experimental observations.^24,28^ The DPD-calculated inter-rod spacing for SDS (i.e., *n* = 0) at room temperature has a value in real units of *r*_S_ = 4.25 nm, at both concentrations trialed of 40 and 50%. This is a slight underprediction when compared with experimental observations of 5.0 nm at 40% and 4.7 nm at 50%.^24^ As discussed in Section 3.1, experimentally, we observe a large increase in the viscosity of AES solutions for hexagonal phases with high concentration. This may be due to a decrease in the inter-rod spacing at high concentrations.

For AES at 40% concentration, the inter-rod spacing is calculated as *r*_S_ = 8.97 (in DPD units), which is located in between the result for *n* = 0 (*r*_S_ = 7.52) and *n* = 1 (*r*_S_ = 9.65), at a value which is consistent with the theory that AES periodicity can be interpolated from monodisperse simulations (see eq 17). However, for simulations at 50% AES, the hexagonal phase takes an inter-rod spacing value which is larger than results for both *n* = 0 and *n* = 1. It is unclear from our results why this might be the case.

Relative to the lamellar phase, it is more difficult to create a finite list of the accessible spacing values for a given box size *L*. Although in theory, there exists a large number of varying integers that can satisfy eq 13, in practice, the rods form to keep the magnitude of lattice vectors |*a⃗*| and |*b⃗*| as similar as possible. This is to be expected, as this is what is found experimentally since  minimizes the potential energy. Based on the restrictions provided by eq 13 alone, there are a huge number of possible inter-rod spacings that the unit cell can take. If the restriction is imposed that the difference in length of the vectors defining the unit cell should not be more than a particular cutoff *d*_co_, then a reduced number of available inter-rod spacings *r*_S_ can be obtained. Mathematically, this can be written as
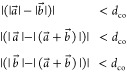
19where vectors *a⃗* and *b⃗* are defined in Figure 4. It is observed in this work that the unit cell never forms with 1.5 < *d*_co_. Therefore, an estimate for the available inter-rod spacings *r*_S_ can be obtained applying the cutoff to *d*_co_ = 1.5. Combining the abovementioned constraint and the restrictions given by eq 13 provides an estimate for the available average inter-rod spacing values in Figure 12.

The underprediction of SDS inter-rod spacing may be a result of the restrictions imposed on *r*_S_ from a finite box size. Although there are other theoretically accessible values of *r*_S_ from unit cell rotation and distortion in the *y*–*z* plane, the application of shear may be causing a degree of alignment in the *y*–*z* plane, as well as in the *x*-dimension. Experimentally, hexagonal phases under shear can exhibit a preference for forming with lattice vector *a⃗* parallel to the shear plane,^56,58^ which is the orientation illustrated in Figure 4. Although it is difficult to quantify the degree to which this is happening in our work (due to the lack of perfect unit cell formation), there is some evidence that the DPD hexagonal phases prefer to take this parallel alignment , as opposed to a perpendicular arrangement . For example, the average inter-rod spacing *r*_S_ = 9.65 is formed in four simulation cases in Figure 12. This value of *r*_S_ is the result of a hexagonal phase which has a unit cell with one of the lattice vectors being equal to 10, which is of significance since the box size *L* = 40 is an integer value of the magnitude of the lattice vector. This means that this unit cell with *r*_S_ = 9.65 describes either a perfect parallel or perfect perpendicular orientation, and both orientations would take the same average value of inter-rod spacing. In all four cases, the simulation takes the parallel arrangement, as opposed to the perpendicular, therefore exhibiting a preference for one orientation over another.

One of the challenges modeling liquid crystals using DPD is the impact of a finite box size. Although these problems can largely be overcome with large enough boxes, this greatly increases the equilibration time of the simulations. It is also difficult to reproduce a perfect hexagonal liquid crystal, due to the need to satisfy the PBCs. It is clear that the number of available inter-rod spacings obtainable in a box size of *L* = 40 is relatively low. In the range 7.5 < *r*_S_ < 11, the number of available values for a box of size *L* = 40 is just 10, while an increase to box size *L* = 50 is found to generate 45 different available values of *r*_S_, when applying the same conditions as those used for Figure 12. However, since the simulation time scales as approximately ∝ *L*^3^ log  (*L*^3^) (scaling is dominated by calculation of electrostatics), an increase in box size from *L* = 40 to *L* = 50 would take over twice as long to complete the same number of iterations.

In summary, the hexagonal phases are the most challenging of the three main phase structures to study. This primarily results from their long equilibration time, and therefore, we resort to the application of shear in order to encourage their formation. The application of shear causes phase alignment, in a way that is in agreement with what is observed experimentally.^56−58^ The hexagonal structures also suffer the most from finite-size effects, leading to our inability to form perfect hexagonal structures. However, we are still able to conclude that the inter-rod spacing is dependent on both the number of ethoxy groups and the concentration, in a similar way to the lamellar phases already discussed.

## 4 Conclusions

In this work, we have established the phase diagram at room temperature for pure SLE_*n*_S surfactants in the range *n* = 0–3. We study the whole of the phase diagram across a large concentration range, where we find good agreement with experimental phase diagrams for AES, SDS,^1^ and SLE_3_S.^2^ In general, the correct type of phase is identified as a function of concentration and ethoxylation, and phase boundary locations are in good agreement with experimental data. In the micellar region of the phase diagram, micelles are relatively spherical until reaching concentrations *c* > 20%. However, for each value of the number of ethoxy beads, there is an “optimal’ aggregation number for which the micelles are most spherical. This spherical aggregation number is found to increase with increasing degree of ethoxylation. With further increase in concentration, we see that the transition from a micellar to a hexagonal phase is rather gradual, taking place through a worm-like micellar phase. In contrast, the hexagonal–lamellar phase transition at a concentration of ≈60% is more abrupt. No cubic phases were identified for SLE_3_S, although these only occur experimentally for a very narrow region of the phase diagram. The lack of cubic phases may be a result of the application of shear in order to induce the hexagonal phase. Hexagonal phase simulation using DPD has been found to be a challenge by many authors, and therefore, applying shear to the system is a practical solution to the problem of equilibration for these systems.

DPD has allowed us to easily vary the ethoxylation and concentration in order to investigate the effect that this has on the periodicity of liquid crystals. This is of interest due to the impact this periodicity has on properties of liquid crystal solutions, such as the viscosity and rate of dissolution. The general behavior observed in this work is consistent with the experimental data available, with the periodicity in liquid crystals increasing when lowering the concentration and increasing the number of ethoxy groups. There is a slight underprediction of the absolute value for the *d*-spacings and inter-rod spacing in DPD when compared with experimental data. This could be a result of a variety of assumptions in the DPD model. One source of error could be the treatment of the electrostatic force, since the effect of charge smearing is relatively untested at high concentrations. The parameterization introduced by Anderson et al.^11^ which is used in this work also does not investigate the effect of bond constants in the harmonic potentials presented in eqs 8 and 9. This could have an influence on the liquid crystal packing at high concentrations.

Underprediction of the mean aggregation number for micellar systems is likely to be, in part, due to the treatment of the electrostatic force, which is also suggested as a potential reason by Anderson et al*.*^11^ Although DPD has been extensively applied to surfactant solutions by a variety of authors, the majority of previous studies focus on the behavior of nonionic surfactants. This results from nonionic surfactants being significantly easier to model, as the long-range electrostatic interaction force does not need to be included. Incorporating the electrostatic force into the DPD method is more difficult than for MD simulations. Since DPD models molecular interactions using soft repulsions, the typical approach of modeling atoms as point charges can cause problems. Therefore, electrostatic repulsions in DPD are typically modeled using a smeared-charge distribution,^11,32,36,60^ as was performed in this paper. This removes the problems that result from treating atoms as point charges; however, the full effect that using a smeared charge has on the resulting behavior is not well studied and requires further research. The difference between experimental data and DPD, as presented in this work, is more pronounced for lower concentration systems, backing up this hypothesis. We also find it of interest that in the work of Peroukidis et al.,^61^ who investigate SDS micelle formation, they find greater discrepancy with experiment data for coarse-grained simulation results when compared to all-atom simulations. Interestingly, our results using DPD for SDS 7% solutions (*N*_agg_ = 44) more closely match their all-atom results (*N*_agg_ = 71) than their coarse-grained case (*N*_agg_ = 157), indicating that out underprediction of aggregation number is not simply due to the act of coarse graining.

The extent to which simulations of monodisperse surfactants can be used to reproduce the behavior of polydisperse surfactants has been investigated in this work. This is of note since the majority of computational work focuses on monodisperse calculations, while the majority of experimental work focuses on polydisperse calculations. For example, Figure 8 shows that for micelles produced by a polydisperse surfactant, the radius of gyration *R*_G_ and the aggregation number *N* of micelles can largely be predicted from interpolation of monodisperse surfactant simulations. Calculations of the lamellar phase *d*-spacing yielded a similar conclusion. An equivalent investigation could not be performed for the inter-rod spacing of the hexagonal phase, due to the limited number of spacings available. However, we present evidence which suggests that it is reasonable to represent polydisperse surfactants using more simple, monodisperse surfactant simulations, which is usually assumed but often not verified.

In summary, DPD has been found to be a useful tool for simulating a system that is difficult to study experimentally. Experimentally, the formulation of pure, single-component SLES systems is difficult, and therefore, there is limited existing literature dedicated to understanding the effect of the degree of ethoxylation on solution behavior. We have shown that the periodicity of liquid crystals is influenced by both the degree of ethoxylation and concentration. Using simulation, we have been able to analyze aspects of micellar solutions and mesophase behavior which are also difficult to measure experimentally, such as micelle shape and phase transition boundaries. In this work, we highlight that DPD simulation can be an effective tool for uncovering aspects of the surfactant solution structure, which is useful for the design of future surfactant-containing products. The simulations conducted in this study for SLES surfactants could be easily extended to other common surfactants, as well as more complex surfactant mixtures. Simulations of mixtures in particular allow for the investigation of new potential formulations, for use in commercial products.

## Supporting Information Available

Examples of the POM imaging used to categorize phase types (PDF)
