# Supplementary material for: Studying the Structure of Sodium Lauryl Ether Sulfate Solutions Using Dissipative Particle Dynamics
Source: J Phys Chem B. 2022 Sep 30;126(40):8058–71. doi: 10.1021/acs.jpcb.2c04329 (PMC9574933; doi:10.1021/acs.jpcb.2c04329)
Supplement: Supplementary file 1 — jp2c04329_si_001.pdf [file jp2c04329_si_001.pdf]

Supporting Information

Studying the Structure of Sodium Lauryl Ether Sulfate (SLES) Solutions Using Dissipative Particle Dynamics

Authors: Rachel L. Hendrikse* [1 2], Andrew E. Bayly[1] and Peter K. Jimack[3]
Affiliations: [1] School of Chemical and Process Engineering, University of Leeds, Leeds, United Kingdom, [2] EPSRC Centre for Doctoral Training in Fluid Dynamics at Leeds, University of Leeds, Leeds, United Kingdom, [3] School of Computing, University of Leeds, Leeds, United Kingdom

Polarised Optical Microscopy for AES Phase Identification

A summary of the different textures identified for different AES concentrations is shown in Table S.1. Structures from POM images are categorised by visual inspection. Concentrations 6.9, 13.2 and 20.1% exhibit no textures, indicating the existence of an isotropic solution. Qualitatively the viscosity of these solutions is relatively low, therefore they will be assigned to the micellar phase. There then exists a large hexagonal region for samples of concentrations from 28% up to 58.6%, which are identifiable from their marble/smoke-like or mosaic textures. A sample of 59.9% displays a transition to lamellar phases, which is identifiable from a more streaked like texture. Further increase in concentration continues to display lamellar textures. A selection of microscopy images at different concentrations is shown in Fig. S1. An interesting observation is that the lamellar phases also display a high degree of alignment at solution/air boundaries, as illustrated in Fig. S2. This helps provide an extra point of identification between the hexagonal and lamellar phases.

Sample Concentration (wt.%)	Appearance
6.9	No Textures
13.2	No Textures
20.1	No Textures
28.0	Hexagonal
34.7	Hexagonal
41.3	Hexagonal
49.7	Hexagonal
52.1	Hexagonal
58.6	Hexagonal
59.9	Lamellar
63.1	Lamellar
70	Lamellar

Table S.1: Identification of the mesophases formed by AES/water solutions at room temperature. Phases identified using POM.

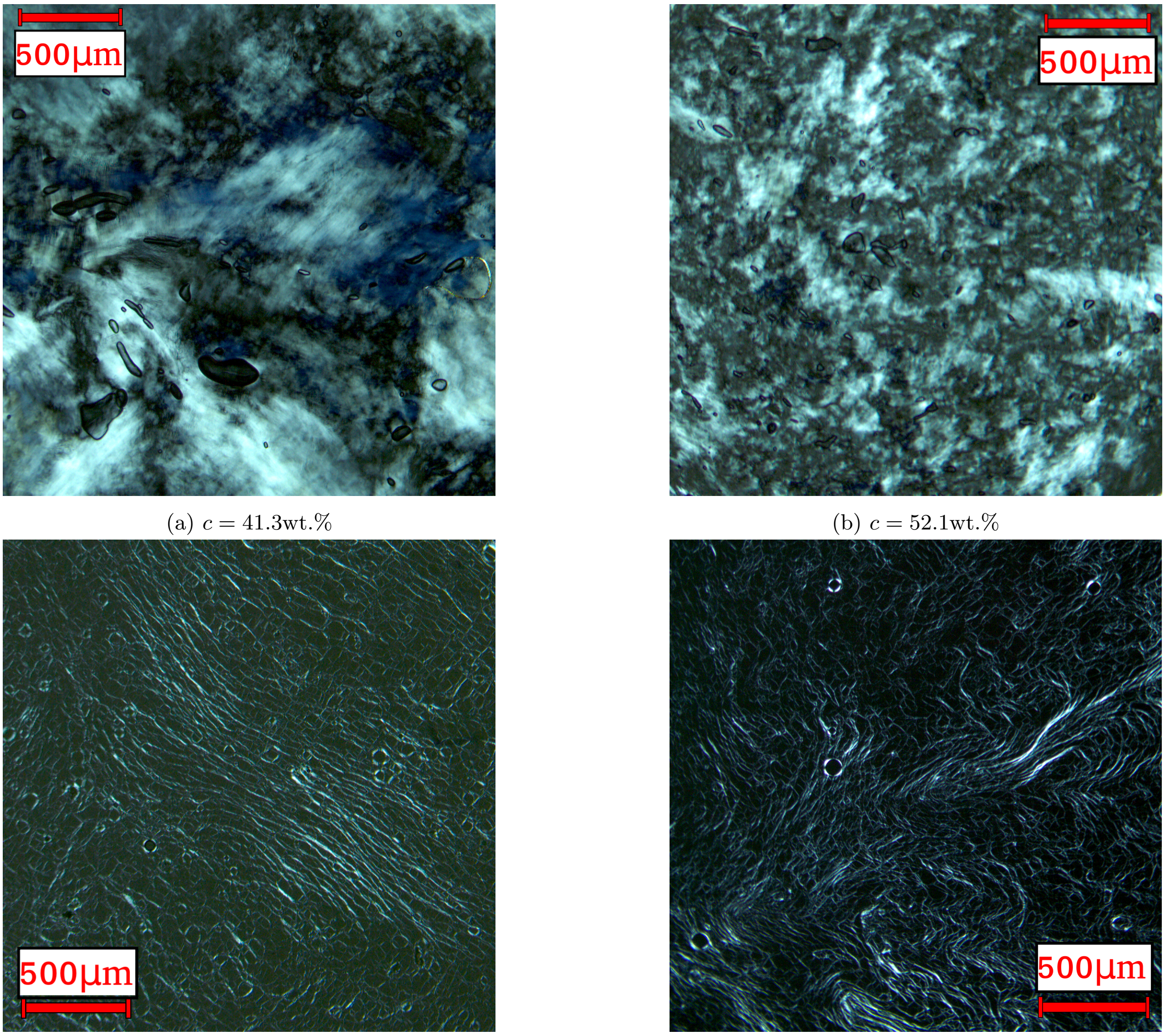

(a) $c = 41.3$wt.% (b) $c = 52.1$wt.%

(c) $c = 59.9$wt.% (d) $c = 70$wt.%

Fig. S1: POM images at 5x magnification of a variety of AES solutions at different concentrations c. Phases identified as hexagonal (a and b) and lamellar (c and d).

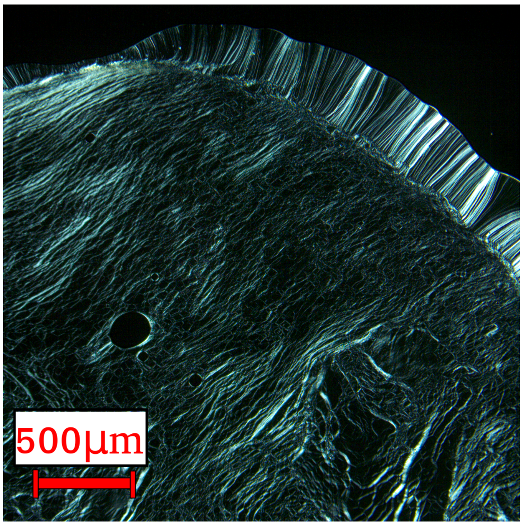

Fig. S2: POM images at 5x magnification of a sample at concentration $c = 63.1$wt.%. Phase is identified at this concentration as lamellar. The region of this image that is completely black is where no solution is present. There is increased phase alignment of the lamellar phase at the phase boundary between the bulk surfactant and the interface with the air.
